# Internal State: Dynamic, Interconnected Communication Loops Distributed Across Body, Brain, and Time

**DOI:** 10.1093/icb/icab101

**Published:** 2021-06-11

**Authors:** Jessleen K Kanwal, Emma Coddington, Rachel Frazer, Daniela Limbania, Grace Turner, Karla J Davila, Michael A Givens, Valarie Williams, Sandeep Robert Datta, Sara Wasserman

**Affiliations:** Division of Biology and Biological Engineering, California Institute of Technology, Pasadena, CA 91125, USA; Department of Biology, Willamette University, Salem, OR 97301, USA; Division of Neurobiology and Behavior, Columbia Universitye, New York, NY 10027, USA; Department of Neuroscience, Wellesley College, Wellesley, MA 02481, USA; Department of Neuroscience, Wellesley College, Wellesley, MA 02481, USA; Department of Biology, Willamette University, Salem, OR 97301, USA; Department of Biology, Willamette University, Salem, OR 97301, USA; Department of Dance, The Ohio State University, Columbus, OH 43210, USA; Department of Neurobiology, Harvard Medical School, Boston, MA 02115, USA; Department of Neuroscience, Wellesley College, Wellesley, MA 02481, USA

## Abstract

Internal state profoundly alters perception and behavior. For example, a starved fly may approach and consume foods that it would otherwise find undesirable. A socially engaged newt may remain engaged in the presence of a predator, whereas a solitary newt would otherwise attempt to escape. Yet, the definition of internal state is fluid and ill-defined. As an interdisciplinary group of scholars spanning five career stages (from undergraduate to full professor) and six academic institutions, we came together in an attempt to provide an operational definition of internal state that could be useful in understanding the behavior and the function of nervous systems, at timescales relevant to the individual. In this perspective, we propose to define internal state through an integrative framework centered on dynamic and interconnected communication loops within and between the body and the brain. This framework is informed by a synthesis of historical and contemporary paradigms used by neurobiologists, ethologists, physiologists, and endocrinologists. We view internal state as composed of both spatially distributed networks (body–brain communication loops), and temporally distributed mechanisms that weave together neural circuits, physiology, and behavior. Given the wide spatial and temporal scales at which internal state operates—and therefore the broad range of scales at which it could be defined—we choose to anchor our definition in the body. Here we focus on studies that highlight body-to-brain signaling; body represented in endocrine signaling, and brain represented in sensory signaling. This integrative framework of internal state potentially unites the disparate paradigms often used by scientists grappling with body–brain interactions. We invite others to join us as we examine approaches and question assumptions to study the underlying mechanisms and temporal dynamics of internal state.

## We begin here

We came together as a group of neuroethologists, a neuroendocrinologist, computational behavioral biologists, a professor of dance, and five undergraduates to articulate an operational framework of internal state. Our collaboration arose during the uncertain times of the Coronavirus (COVID-19) pandemic, as the almost universal shift to remote work allowed us to connect across many locations and time zones. Altogether, we implemented an integrative and iterative approach that enabled a synthetic framework of internal state to emerge. We intentionally refer to ourselves using plural personal pronouns (we, our, and us) as we share not only our synthesis of internal state but also aspects of our co-creative process. In this Perspective, we use internal state to refer to the set of cellular, metabolic, and systems-level activities that modify how sensory information is dynamically represented and communicated between the body and the brain. We invite you to join us on our journey and ongoing discussions as we explore internal state through the lens of history, recent breakthroughs, and future challenges.

## A brief history of internal state: perspectives from the body and brain

The current notion of internal state began with the concepts of homeostasis and interieur milieu (Cannon and Rosenberg 1932; Holmes 1986; Cross and Albury 1987; Gross 1998). Homeostasis is the self-regulating process by which biological systems maintain stability while adjusting to changing external conditions (Cannon and Rosenberg 1932; Billman 2020). Homeostasis itself was built on the concept of interieur milieu, which refers to the idea that the chemical composition of the internal environment (i.e., interstitial fluids) is actively maintained around stable settings and that this stability is a prerequisite for the development of a complex nervous system (Gross 1998). These ideas have their roots in the ancient concepts of humors and balance, two frameworks used in medicine dating back to at least 6–1 Before the Common Era (BCE) (Gross 1998; Cantor 2002; Craik 2009; Köhle 2016).

**Fig. 1.**
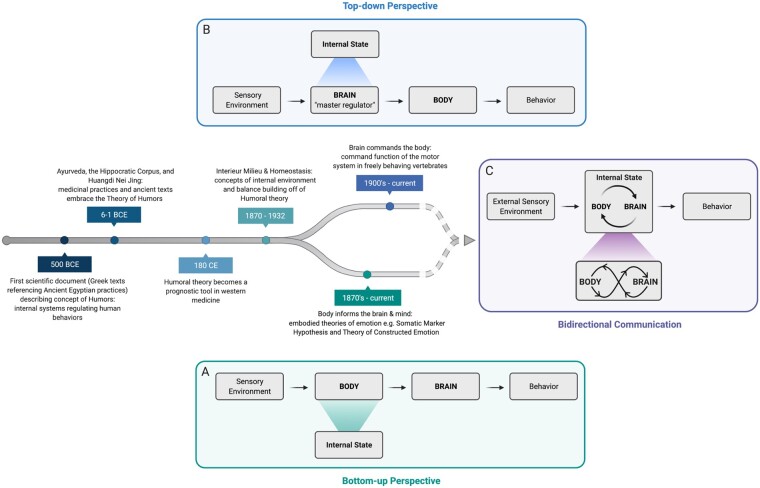
Historical contributions and conceptual frameworks shaping current concepts of internal state. The notion of internal state dates back to the concept of humors, or the bodily fluids thought to modulate human behavior and health, as referenced in the ancient texts of Egyptian, East Asian, and Greek philosophers. Building on the concept of humors, Western physiologists coined the terms interior milieu (“internal environment”) in the late 1800’s and homeostasis in the 1930’s, used to refer to the internal fluids and steady-state conditions important for survival. Thereafter, two major perspectives predominate the internal state literature: (**A**) a bottom-up perspective focused on the internal physiology of the body and information flow from the body-to-brain and (**B**) a top-down perspective focused on internal state representation in the brain and information flow from the brain-to-body. Knowledge gained from these approaches combined with modern tools now supports an integrated framework of internal state that seeks to understand the bidirectional communication pathways between the body and brain (**C**). An infinity loop between body and brain represents internal state as the dynamic crosstalk both between and within these systems as well as their associated set of cellular, circuit, and systems level activities.

The concept of humors includes systems of medicine based in India (ayurvedic medicine; Patwardhan 2016; Jaiswal and Williams 2017) and China (Huangdi Nei Jing; Liu 1988; Craik 2009), as well as the European equivalent in the form of the Hippocratic corpus, a 60-70 volume set of work of which one volume was dedicated to humors and balance (Cantor 2002; Iniesta 2011). Traditional systems of medicine from India and China were implementing humors as diagnostic health tools long before they were included in Western canon (Iniesta 2011; Fig. 1). What is striking to us is that these foundational texts are thought to reference even older, image-based texts depicting concepts equivalent to humors from Egyptian practices dating back to 5000–2000 BCE (Freeman 1983; Billman 2020). Most of these historical perspectives assume a bottom-up information flow in which the body informs the mind.

This historical perspective in some ways contradicts the current dominant perspective in neuroscience and psychology—that the brain commands the body. There is a wealth of evidence from two centuries of psychology and neuroscience demonstrating that neural circuits are organized hierarchically to control muscle movement and physiology within the body, and that in some real sense the main outputs of the brain are internal and external behaviors. Contemporary studies have shown that multiple top-down pathways modify various aspects of peripheral physiology (Fig. 1 timeline; Armstrong 1986; Rossignol et al. 2006; Grillner et al. 2008; Anderson 2016). These discoveries, among many others, have contributed to a feed-forward, top-down view in which the brain has primacy over the body. Although top-down and bottom-up perspectives are compatible with each other (and indeed, as we argue, likely essential to understanding the fullness of influence of internal state upon the brain), social and historical trends have artificially divided researchers concerned with brain function from those exploring the homeostatic regulation of the body, although many are calling for a more integrative view of this problem (Barrett 2006; Damasio and Carvalho 2013; Buzsáki 2019; LeDoux 2020; Fig. 1).

## A new synthetic framework

**Fig. 2.**
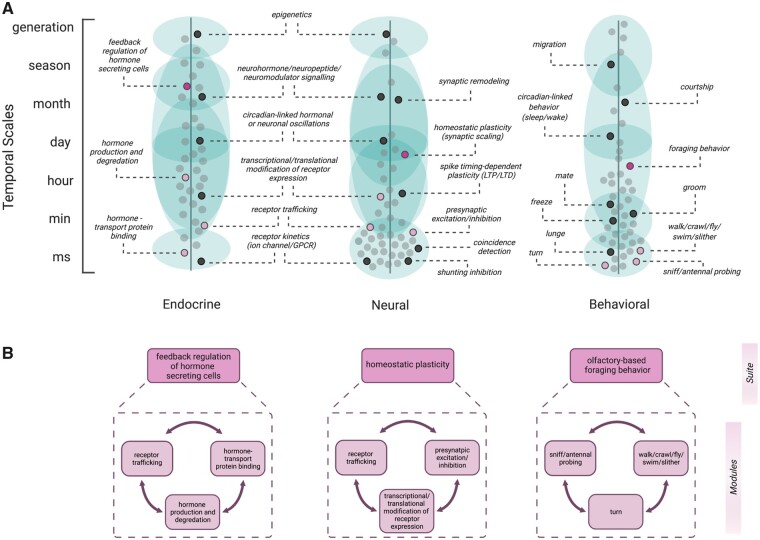
Timescales of endocrine, neural, and behavioral modules. Representation for various modules of integration and communication across temporal scales along three spatial dimensions: endocrine, neural, and behavior. A modular approach to describe discrete behavioral units that together perform a behavioral function, like the actions that contribute to a fixed action pattern or the motions that contribute to a courtship dance, is often referred to as syllables of behavior, composed of smaller discrete units often termed modules. We extend the use of the terms syllable and module to represent the modular approach scientists have toward discrete mechanisms of action that are visible within the endocrine and neural systems. Together, sequences of these mechanisms/modules take place as the body and brain communicate the internal needs of the animal and regulate behavioral output accordingly. (**A**) Dots represent examples of modular endocrine and/or neural mechanisms, or units of behavior. Pink dots highlight examples of discrete mechanistic or behavioral modules that can work in combination, leading to changes that are either longer in duration and/or that occur on completely different timescales. Green shaded regions represent the variation and potential flexibility in onset and duration of labeled mechanisms and behaviors. The green envelopes are not comprehensive but serve to highlight the variation observable within an individual across different contexts and/or across different species. We represent this ambiguity and flexibility by the extent and overlap of the shaded green envelope(s) that a particular pink dot is associated with. For example, along the neural axis, homeostatic plasticity is traditionally examined within a particular timescale due to tool availability and the necessity to constrain research questions. Here, we represent homeostatic plasticity being initiated and extending from hours to days; however, these mechanisms can have an onset within minutes and a duration that changes across seasons and lifetimes. (**B**) We offer three examples of theoretical suites within each axis: endocrine, neural, and behavior. Each suite is composed of three mechanistic modules. For example, homeostatic plasticity can arise due to activity-dependent trafficking of AMPA glutamate receptors to the synaptic membrane. The relatively short timescale changes in receptor translation and trafficking along with electric stimulation via presynaptic excitation/inhibition together may lead to long-term plasticity changes. Although homeostatic plasticity is depicted as a suite with modular components in this example, it can also be a module within a suite in other contexts (such as within synaptic remodeling).

In this section, we articulate a framework that describes internal state as integrated top-down and bottom-up communication loops between the body and brain. In doing this, we render explicit that which is often left implicit: that multidirectional body–brain communications loops compose internal state. Furthermore, this framework places spatially distributed body and brain communication loops (Fig. 1C) on a distributed temporal scale (Fig. 2A). Typically, any one research project is constrained in studying mechanisms or behavior on a few specific temporal scales: milliseconds to minutes, minutes to hours, days to months, sometimes lifetimes, or occasionally over generations. By necessity, projects tend to focus on one space–time mechanism, and often cannot attend to the myriad of ways in which other scales are layered within and underneath, like the hidden structures of a house. Below, we evidence how this framework can reveal a more extensive landscape of mechanisms underlying behavior. Like any effective working model, this framework allows us to identify gaps in our knowledge, and discuss dynamic mechanisms enabling nuanced and flexible behaviors.

### Part 1: Integrating top-down and bottom-up perspectives on internal state

We began by broadly defining the bottom-up and top-down perspectives of internal state. The bottom-up perspective posits that internal state corresponds to the physiological and metabolic changes sensed, filtered, and integrated by the body. These changes are relayed from the body to the brain to coordinate appropriate behavioral output. From this body-centric view, the primary function of the brain is to regulate and respond to signals originating from within the body (Fig. 1A). The alternative top-down perspective asserts that the brain acts as a master regulator, responsible for processing, filtering, and integrating external sensory information from the environment with internal sensory inputs from the body. Further, the top-down perspective rests on the assumption that the brain commands and coordinates changes in the body that allow an animal to perform appropriate state-dependent behaviors (Fig. 1B). What would a framework incorporating both of these notions look like?

We represent internal state as an infinity loop connection between the body and brain (Fig. 1C). The infinity loop signifies that there is no clear starting or ending point, and therefore no master controller, when it comes to the processes that determine internal states and drive behavior. Further, the infinity loop indicates that in addition to bidirectional flow of information between the body and brain, there are also multiple feedback loops within the body and brain, as evidenced from studies across a diverse array of species (Hartenstein 2006; Droujinine and Perrimon 2016; Nässel and Zandawala 2020; Norris and Carr 2020). We ask you, the reader, to explicitly broaden this framework to incorporate information distributed spatially across the body and brain, including but not limited to muscles, bones, connective tissues, viscera, immune, and endocrine glands.

### Body, brain, and the bridges and boundaries between

To explore mechanistic questions about internal state, we quickly realized that we had to come to a common understanding of what distinguishes the body and brain. Up until this point, we have intentionally left body and brain undefined. Take a moment to consider if or where you place a boundary between body and brain. From our rich conversations, we realized that some of us operationalize the brain as including all nervous system structures, including peripheral sensory receptors, while the body is everything else. However, this distinction begins to blur when we consider peripheral sensory receptors located in internal organs, or the more distributed nature of the nervous system of invertebrate species such as worms, jellyfish, and octopuses. In contrast, others consider the brain as everything that lies along the central axis of the body—including invertebrate ganglionic structures linked via a nerve cord as well as the vertebrate brain and spinal cord. Under this construct, the body includes most if not all sensory organs, receptors, neural net organs (i.e., heart and gut), and even peripheral autonomic nervous system ganglia.

This said, the boundary between body and brain is fluid; literally, located in interstitial space, and metaphorically, shifting as needed. The biological basis of these boundaries spans a large range of dynamic structures and systems that connect, communicate, and coordinate function. These structures at the interface include but are not limited to the lymphatic system, glymphatic system, meninges, blood vasculature, choroid plexus, glial cells, and the skin (Paus et al. 2006; Chen and Lyga 2014; Jessen et al. 2015; Weller et al. 2018; Wilton et al. 2019; Decimo et al. 2020; Kaplan et al. 2020; Thouvenin et al. 2020; Saloman et al. 2020). Many of these boundaries between body and brain are composed of physical connectors and filters, such as the vessels that make up the blood or lymphatic vasculature, as well as fluids, such as the extracellular, lymphatic, and cerebrospinal fluids.

For the purpose of this perspective, we use the term brain to refer to the peripheral and central nervous system—from sensory receptors to motor output. In contrast, the body includes all organs and fluids outside the brain, including but not limited to the immune, endocrine, gastrointestinal, cardiovascular, waste-management, muscle, microbial system, and skeletal systems. We recognize that these constructs dividing up the body and brain are necessitated by the mechanistic questions examined, language available, and the existence of and accessibility to tools. Additionally, we found that such compartmentalization aided in our review of past literature and motivated the development of our framework.

We suggest that internal state arises through a distributed network of pathways composed of the amorphous bridges, between body and brain, as described above. These pathways are degenerate (Tononi et al. 1999; Edelman and Gally 2001; Sajid et al. 2020), resulting in all or some of the organ systems working together to maintain a responsive and relatively stable internal environment. Furthermore, there are many ways in which internal state is established and regulated by external state in animal systems: including but not limited to natural rhythms (circadian and seasonal) and exteroceptive sensory input. These processes all occur on different timescales and recruit or impact iterative internal feedback and feedforward loops (Fig. 1C).

### Part 2: A temporally integrated framework of internal state

Technology has enabled and constrained most neuroendocrine and neuroethological studies to mechanisms that operate within a limited timeframe, however, behavior operates over many timescales. We suggest that physiological systems can be categorized into shorter timescale modules of mechanisms that work together to coordinate longer time scale changes in the body and brain. This applies an ethological approach to understanding physiology.

Ethologists have discretized behaviors as sequences of smaller functional units, which are often referred to as modules (Box 1). Tinbergen developed a specific method by which to categorize behaviors across space and time (Tinbergen 1951). Modules are discrete, stereotyped, and reused units of behavior; this definition of behavioral module is agnostic to timescale. For instance, a module could be a territorial behavior that extends across seasons or a feeding behavior constrained to a few minutes in the day. However, different types of modules are often placed into sequences that compose macroscopic behaviors and are therefore organized at a specific timescale. The behavioral modules that make up fly courtship evolve on the seconds-long timescale; the modules that make up the circadian rhythm—wake and sleep—each last ∼12 h in a typical mammal. Furthermore, as should be obvious from these two examples, behavior is often organized hierarchically, and as such, different modules that are organized at different timescales co-exist and influence each other.

Both supervised and unsupervised machine learning approaches are rapidly improving our ability to identify and characterize behavioral modules at different timescales. Recent work in unsupervised machine learning has identified a set of sub-second behavioral modules that are jointly defined based upon their repeated and stereotyped expression (a prerequisite for any behavioral module) and the sequence in which they are observed to occur over time. Given the grammar-like organization of these fast modules of stereotyped movement—and the intermediate level of the behavioral hierarchy in which they sit—such modules are referred to as behavioral syllables (although similar fast units of action have been referred to alternately as movemes and motifs) (Anderson and Perona 2014; Datta et al. 2019). The utility of considering behavior as being built from modules is that it reveals predictable variations in syllable sequences (and at longer timescales, module sequences). As a consequence, syllables can be used to test the hypothesis that internal state modifies external state by modulating the frequency of, transition between, and order of syllables. It is clear from this ongoing body of work that behavior is much higher dimensional than previously appreciated, and that capturing and organizing this high-dimensional information is essential for understanding the intersection between body and brain.

We extend the concept of modules from ethology to include both neural and physiological mechanisms (Fig. 2). This enables us to visualize the multidimensional nature of different mechanisms (modules) occurring across space and time that can contribute to any particular function. For instance, within the endocrine loop, feedback regulation of hormone secretion is a function, and can be parsed into at least three more discrete and measurable mechanistic modules occurring on shorter timescales that together contribute to a seamless functional output; auto-regulation of hormone secretion is composed of receptor trafficking, hormone-transport protein binding, and hormone degradation (Fig. 2B). Within the neural loop, three modules that contribute to homeostatic plasticity include: presynaptic excitation/inhibition, transcriptional/translational modification of receptor expression, and receptor trafficking (Fig. 2B). When we consider these functions to be composed of modules, we can immediately observe points in time where crosstalk between endocrine and neural mechanisms can occur (i.e., receptor trafficking). This framework can also reveal how internal state impacts a particular behavior via mechanisms occurring at timescales beyond the range of those captured by any one experiment or project. Thus, the contributions of long timescale influences, such as generationally inherited information and seasonally experienced events, can be layered into short timescale decisions about which behavioral modules to express.

In summary, our framework incorporates three key perspectives. The first is to recognize that the body and brain ultimately function as a single unit, where internal state is an emergent property of both body and brain physiological states. The second is to expand the dimensionality of internal state by placing the underlying mechanisms along a temporal axis. The third is to consider how discretized mechanisms weave together across time to inform internal state and drive flexible behavior.

### The scope of our dive into this framework

Constrained by space and time ourselves, we elected to focus on the role of bottom-up, body-to-brain communication in establishing internal state. We focus our perspective further, by exploring one aspect of the body, the endocrine system, and one aspect of the brain, sensory reception and perception. The endocrine system is one of the key dynamic mechanisms by which organ systems communicate with each other—for instance, via hormones traveling via the blood, lymph, or hemolymph.

While we recognize that sensory and endocrine systems perform many different important functions for an organism, one of the vital roles of these systems is to guide an animal’s behavior toward acquiring basic needs (Maslow 1943). Deprivation of these needs leads to internal state changes, such as hunger, fear, and anxiety, and these changes prompt robust and measurable compensatory processes that include behavioral changes. For instance, hungry animals may increase foraging behaviors and decrease sleep, in order to help the body regain blood sugar or other nutrient levels necessary for survival.

**Fig. 3.**
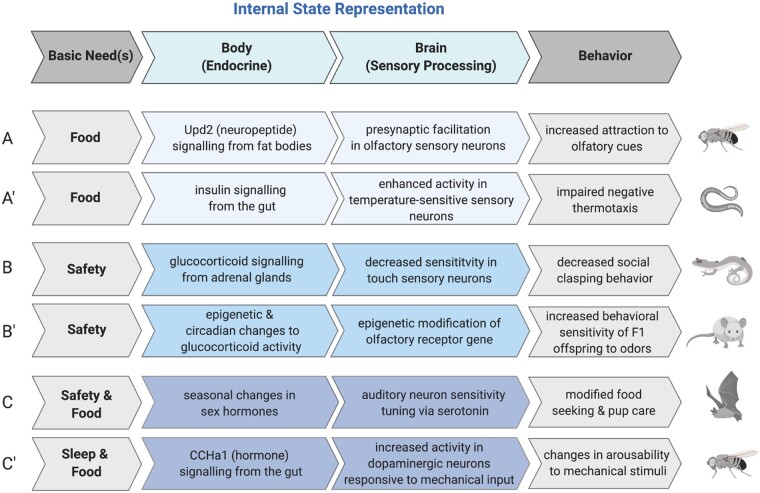
Absence of basic needs detected by endocrine mechanisms in the body modulate sensory processing in the brain to drive flexible behavior. Organisms must be able to represent and communicate the availability, or lack thereof, of basic needs in the environment to drive contextually appropriate sensory processing and behavior. Here, we outline example endocrine and sensory processing mechanisms that work in concert to promote flexible behavior across model and non-traditional model organisms. We note that these mechanisms have been simplified and may not represent all mechanisms at play. Example body-to-brain signaling and resulting behavioral modification(s) in response to the absence of food in (**A**) the fruit fly, *D. melanogaster*, (**A'**) the nematode, *C. elegans*; absence of (**B**) acute safety in the newt, *Taricha granulosa*, (**B'**) chronic safety in rodents; absence of multiple needs in (**C**) the big brown bat (*Eptesicus fuscus)*, and (**C'**) the fruit fly, *D. melanogaster.*

Most physiological responses to basic needs like hunger, sleep, and safety require multi-organ interactions. Inspired by Krogh’s principle: “For a large number of problems there will be some animal of choice, or a few such animals, on which it can be most conveniently studied” (Krogh 1929; Miller et al. 2019; Jourjine and Hoekstra 2021), we examine examples of body-to-brain signaling used to communicate a change in the availability of food and/or safety across selected model and non-traditional model organisms (Fig. 3).

In an effort to explore how internal state coordinates behaviors, we focus on the body-to-brain direction of communication. We constrained our search to endocrine and sensory systems and a few basic needs over a range of timescales. Our hope is that the examples below, some of which have not been fully elucidated, will identify future research questions and encourage researchers across disciplines, to consider how signaling within and from the body contributes to internal state and behavior. This signaling occurs over timescales that might differ from those traditionally used to measure activity at the neural or behavioral levels and will likely reveal novel and exciting new mechanisms of communication between the body and the brain.

## Contextualizing through our framework

### How does the body encode and communicate absence of food to drive flexible neural signaling and behavior?

To illustrate the importance of body-to-brain communication, we highlight how the disruption or absence of food modulates sensory processing and behavior. Food is a basic need essential for the growth, development, energetics, and survival of all organisms. Nutrient-sensing organs throughout the body, including the gastrointestinal tract, pancreas, and fat cells, continuously monitor and regulate nutrient availability and absorption. Signaling messengers of the endocrine system, such as hormones and neuropeptides, are released from nutrient-sensing body organs and travel via the bloodstream to the brain and sensory periphery to alter perception and modify foraging and feeding behaviors (Sengupta 2013; Stowers and Liberles 2016).

The degree to which foraging behaviors change in hungry organisms depends, in part, on the duration for which animals are food deprived or starved. Food deprivation, on the order of hours to days, can lead to a striking perceptual switch in the valence of chemosensory stimuli, shifting animal behavior in response to certain odors from aversion to attraction (Root Cory et al. 2011; Sengupta 2013; Vogt et al. 2021). For instance, in walking assays, satiated adult flies find CO_2_ and high concentrations of the vinegar odor aversive; however, starved flies find the same concentrations of these odors attractive (Root Cory et al. 2011; Bräcker et al. 2013; Siju et al. 2014; Ko et al. 2015). Starvation can also enhance gustatory and olfactory sensitivity, allowing for increased attraction to certain tastes such as sugar or enhanced detection of low odor concentrations (Marella et al. 2012; Inagaki et al. 2014). Additionally, starvation regulates thermosensory behaviors, altering an organism’s foraging strategy, baseline temperature preference, or thermoregulatory behaviors such as shivering (Tan and Knight 2018; Takeishi et al. 2020a, 2020b). In the context of hunger, we have elected to focus on a few studies using invertebrate model systems that have begun to elucidate how key endocrine and neural players and mechanisms coordinate changes in behavior over varying timescales (Root Cory et al. 2011; Ko et al. 2015; Takeishi et al. 2020a, 2020b; Fig. 3A and A').

### Body-to-brain communication

One of the most essential endocrine signaling molecules involved in orchestrating the body’s acute hunger response is insulin. In the worm *Caenorhabditis elegans*, food deprivation leads to insulin release from the gut, which in turn activates a bilateral pair of peripheral sensory neurons (called AWC) that respond to temperature (Fig. 3A'; Takeishi et al. 2020b). Temperature-mediated responses in satiated worms typically rely on the core-thermotaxis circuit (mediated by AFD and AIY neurons), which promotes a sequence of forward crawling, turn, and reversal behaviors that allow the worm to navigate to the most-favorable temperature region in their environment (Mori and Ohshima 1995; Takeishi et al. 2020a). However, when worms are food deprived, this normal thermotaxis response is disrupted due to the recruitment of a parallel pathway mediated by the AWC neurons. AWC and the downstream circuitry instead promote increased turn and reversal behaviors, as worms search for food instead of thermotaxing to their preferred temperature (Takeishi, et al. 2020b). Thus, a worm’s satiety state can lead to insulin-mediated activation of sensory neurons, which in turn drastically modulates the behavioral response of the animal. Insulin orchestrated body-to-brain signaling can also stem from fat bodies, as is the case in starved (18–45 h) *Drosophila melanogaster* fruit flies (Umezaki et al. 2018). Although the exact mechanism remains unclear, insulin signaling from the fat body shifts the response properties of warm-sensing sensory neurons, driving these cells to peak at lower temperatures in hungry flies. This shift at the sensory neuron level results in behavioral changes such that hungry flies prefer a lower baseline temperature compared to their well-fed counterparts.

Often, multiple endocrine signaling molecules work together to communicate and alter sensory processing in response to internal state changes associated with hunger. For instance, starved flies experience decreased fat body secretion of Upd2, a functional homolog of the mammalian Leptin peptide (Rajan and Perrimon 2012; Lin et al. 2019). Low Upd2 levels indirectly inhibit insulin release from insulin producing cells in the brain. The decreased insulin in the brain leads to presynaptic facilitation (increased signal strength) of olfactory receptor neurons (ORNs), via up- or downregulation of transcription for certain neuropeptide receptors expressed at ORN axon terminals (Root Cory et al. 2011; Jouandet and Gallio 2015; Ko et al. 2015). This cascade of signaling from body-to-brain increases olfactory sensitivity and enables hungry flies to detect low odor concentrations or approach typically aversive odors (Fig. 3A).

### Timescales that weave endocrine, neural, and behavioral modulation

**Fig. 4.**
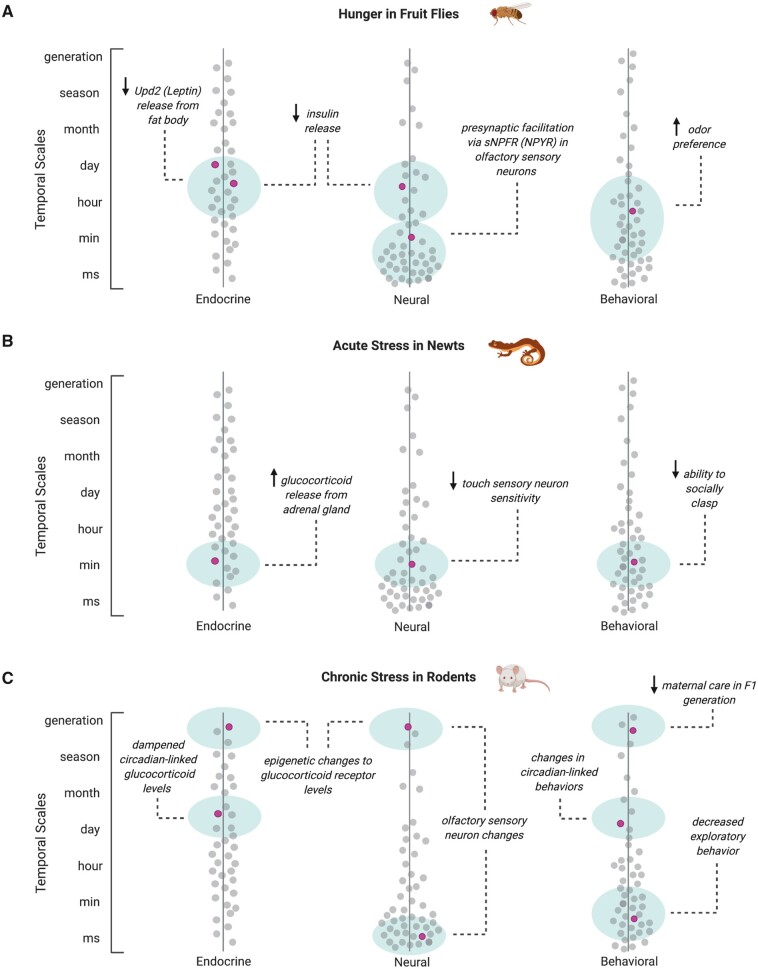
Timescales of endocrine and neural modulation and associated behavioral responses linked to internal state-dependent changes. Endocrine, neural, and behavioral changes associated with internal states can vary from milliseconds (momentary) to lifetimes (generational). Here, we show the timescale and neural, endocrine, and behavioral changes that occur for three examples when animals are deprived of food or safety. Changes shown are associated with (**A**) food deprivation in the fruit fly, *D. melanogaster*; (**B**) challenge to safety in the roughskin newt, *T. granulosa*, during a discrete and acute challenge; and (**C**) lack of safety in rodents during chronic stress. Along the endocrine and neural axes, pink dots indicate an internal state-associated mechanism/process that changes in the body or brain, respectively. Along the behavioral axis, pink dots indicate a change in behavior upon deprivation of a basic need. Shaded green regions represent estimated variation in onset and duration of identified mechanism or behavior, as different studies indicate changes over a range of time.

Interestingly, the timescale of changes along the endocrine, neural, and behavioral dimensions vary in the above example of hungry flies (Fig. 4A). Changes in Upd2 hormone levels occur on the order of days, whereas presynaptic facilitation in ORNs occurs on the shorter time scale of hours. Further, behavioral changes in odor preference can be observed within minutes when comparing hungry to well-fed flies. So, how do these mechanisms work in concert to mediate behavioral changes as the fly’s hunger state is altered? The answer to this question remains unclear, in part because of the disparate timescales at which endocrine, neural, and behavioral changes emerge. Measuring the temporal dynamics of endocrine signals, such as Upd2, with high resolution is a challenge due to the lack of tools available to continuously monitor most endocrine signals. Neural signals, on the other hand, can be measured at a very high temporal resolution using electrodes or optical imaging techniques. Future progress in understanding body–brain communication, and therefore internal state, will greatly benefit from the development of new tools to measure the dynamics of signaling molecules in the body across an array of timescales that bridge those relevant to neurobiology and physiology.

The examples above demonstrate how examining the response of and communication between the body and brain, can uncover a new understanding of an animal’s representation of internal state. By looking at the internal state of the body (i.e., endocrine mechanisms), studies are beginning to uncover how signaling within the body can directly impact sensory neurons. This, in turn, changes our functional understanding of how entire sensory circuits detect and encode stimuli. Furthermore, comparing the timescale of modules of endocrine and neural mechanisms along with those of behavior opens the door for further research questions about whether there may be additional mechanisms at play throughout the temporal scale.

### How does the body encode and communicate challenges to safety to drive flexible neural signaling and behavior?

In this perspective, we elected to examine safety as the second basic need because physiological and neural mechanisms facilitating context-specific responses to safety violations have been documented spanning the entire temporal framework we examine here—from milliseconds to generational timescales. Anticipatory or approaching threats and active violations to safety are termed as “stressors.” Internal states associated with stress will elicit one or more of an array of emotions and defensive behaviors, intended to re-establish safety. For example, anticipatory and current threats to safety and sovereignty trigger anxiety and fear, respectively, the behavioral consequences of which are freeze, fight, or flight responses. Ethological analysis reveals that fear and anxiety will lead to a more nuanced and sequenced suite of distinct defensive behaviors, calibrated to the salience, and proximity of the threat (Endler 1991; Caro 2005). Each behavior in a given defensive sequence will have a species-specific and context-specific manifestation and can be considered as behavioral modules (freeze, fight, flight, tonic immobility, and appeasement)—each composed of some unique and/or common smaller components of movements (Box 1). Animals will employ a cascade of these behaviors in quick succession to enact a behavioral ensemble such as defensive behavior; the timing and signature predictively indicating the specific context and valence of threat. Classical neuroethological work studying defensive behaviors in insects has documented a variety of conditions under which behaviors such as tonic immobility (death feigning or thanatosis) and appeasement are employed as successful defensive behaviors. Conditions include, but are not limited to: avoiding aggressive workers (van Veen et al. 1999), females avoiding male mating attempts and harassment (Dennis and Lavigne 1976), and avoiding predation (Miyatake et al. 2004). This work has indirectly informed current conversations in behavioral and evolutionary ecology (Humphreys and Ruxton 2018; Konishi et al. 2020) and the world of trauma therapy (Keltner et al. 1997; Marx et al. 2008; Humphreys and Ruxton 2018).

### Timescales that weave endocrine, neural, and behavioral modulation

Stress researchers often operationally distinguish between acute and chronic stress conditions. Acute stress refers to rapid responses to an immediate threat, with onsets occurring within seconds and physiological and neural responses affording homeostasis and safety within minutes. Chronic stress is less concrete and can refer to long-term unmitigated stress, to a single untreated traumatic event, or to small stressors that recur on the day-to-lifetime timescale. Challenges to long-term health arise when stressors and subsequent stress-responses run unchecked and unmitigated for long periods of time; often termed chronic stress and resulting in a high disease-potential state (McEwen and Gianaros 2011; Ramsay and Woods 2014; Schulkin and Sterling 2019). It is recognized that the reflexive behavioral responses to acute threats are evolutionarily conserved across the animal kingdom and are highly adaptive in a wide variety of acute and chronic situations. Ultimately, all basic needs will trigger chronic stress responses if any basic need, such as food, water, or safety, is sufficiently unavailable for long enough.

Internal states associated with acute stress are associated with a continuum of physiological responses including increased heart and breathing rate(s), pulse pressure, and blood glucose levels, facilitating an increased metabolic rate. These changes are coincident with decreased gut motility, and extreme acute stress can result in loss of smooth muscle tone resulting in urination and defecation. These physiological changes occur very rapidly, and the amplitude of responses is believed to correspond to the urgency and danger-level of the stressor. Interestingly, these immediate physiological changes do not require input from the central nervous system, although the central nervous system does provide coordination and sustained evaluation of the situation. Furthermore, these changes are accompanied by changes in endocrine and neuroendocrine signaling specific to the threat. Vertebrate endocrine signaling associated with stress typically involves (1) increased circulating levels of epinephrine, a catecholamine hormone synthesized and released by cells in the adrenal medulla, and (2) glucocorticoids (cortisol and corticosterone), a steroid hormone synthesized and released by cells in the adrenal cortex. In both cases, the adrenal glands are involved, glands that are located on top of the kidneys and are responsible for supplying animal bodies with a host of hormones important for homeostasis. Information about the state of the viscera (heart and guts) and endocrine signaling collectively informs the internal state of the animal, which in turn, will inform and result in context-specific behavioral outcomes.

Acute threats occurring within the timeframe of seconds to minutes will result in immediate engagement of multiple and potentially cascading neural and endocrine mechanisms. For instance, three distinct pathways by which acute threats can impact behavior include: (1) sympathetic activity elevated during arousal states associated with stress or excitement, results in widespread changes in cortical brain activity (Özbay et al. 2019); (2) withdrawal reflex arcs which effectively remove body limbs from immediate physical harm (sharp objects or a flame); and (3) upregulated neuroendocrine hypothalamic–pituitary–adrenal (HPA) axis pathways result in the elevation of stress hormones released from the pituitary gland (not addressed here) and adrenal gland—epinephrine, corticosteroids, and mineralocorticoids. Each of these hormones elicits body and brain changes that align metabolic and behavioral responses, designed to bring the animal back to a space that is safe. For an expansion on actions of hormones in this context see any behavioral neuroendocrinology textbook. (e.g., Komisaruk and Gabriela 2020).

There are nuanced differences of impact among the hormones resulting from their unique stoichiometry and chemistry, receptor identities, functions, and locations. Pertinent to this discussion, steroid hormones can function on every timescale that neurotransmitters function and then some (Fig. 2), fundamentally altering brain pathways engaged and sensory processing. Specific to safety, on the fastest end of our temporal scale—an acute challenge to safety (acute stress) results in rapid changes to internal state including elevation of stress-steroids (corticosteroid) and sensorimotor processing, and consequently rapid changes in behaviors. For example, social clasping is rapidly suppressed in newts (Moore and Miller 1984). Clasping is an essential behavior module used by female newts to clasp sticks while laying eggs, used by males to clasp females during courtship, and used by all newts when engaging in post-feeding interactions with each other; clasping involves bilateral flexion of both the fore and hind limbs for a flexible period of time. In rough-skin newts, plasma levels of corticosteroids are elevated within two minutes of experiencing an acute stress (Coddington et al. 2007). Corticosteroids go on to suppress spontaneous activity and sensory responsiveness of touch reception measured in the brainstem and spinal cord within minutes (Rose and Moore 2002; Lewis and Rose 2003), an action that means that stress hormones can literally reduce touch sensitivity. (Figs. 3B and 4B) The interaction of stress, stress-induced hormones (corticosterone), and reduced touch-sensitivity renders newts less able to engage in social clasping for a period of 30–45 min, fundamentally shifting the behavior away from social and toward defensive functions.

Research from rats, mice, and newts has revealed that corticosteroids also promote defensive behaviors within minutes by upregulating cannabinoid signaling in the hypothalamus (Evanson et al. 2010; Tasker and Herman 2011) and brainstem (Coddington 2017). While there is evidence for corticosteroids to modify intrinsic properties of neurons (Duvarci and Denis 2007), in brainstem cells the predominant effect is to modify the rate and volume of receptor-mediated endocytosis events (Davis et al. 2015). We recognize that the tools needed to reveal these effects are expensive and distinct. Furthermore, these mechanisms of action occur on timeframes quite different compared to classical synaptic biophysical approaches. Therefore, practices that encourage a broad mindset, effective collaboration, and access to varied tools are required to reveal a more inclusive suite of mechanisms that might be involved in mediating behavioral responses to stress, or to any state-dependent behavior.

At the other end of our temporal scale (Figs. 3B' and 3C) sits chronic stress. Studies have revealed the impacts of early social experience on fear circuitry and behavior, which can result in changes that are inherited by subsequent generations. Removal of care at critical periods in a rodent’s infancy results in long-term changes to corticosteroid receptor function in the hippocampus and HPA-axis responsiveness to stress (Champagne 2008). These actions can cause generational patterns of neglect in progeny where the subsequent generations (F1 and F2) treat their pups in similarly neglectful ways (Champagne 2008). It is also important to recognize the plasticity of this effect; the impact of removing the mother is reversible if the pups are adopted by nurturing mothers (Champagne 2008). The impact of losing the mother and not offering an adoptive replacement, however, has long-term ramifications on hippocampal-mediated spatial orienting and learning behaviors (Prakash et al. 2006), flattens the HPA-axis hormone cascade, and increases the sensitivity of behavioral responses to acute stress coincident with enhanced sensitivity to glucocorticoids (Liu et al. 1997). One of the mechanisms involved in mediating this long-term cascade of impacts is through long-term modifications of glucocorticoid receptor expression and function in the hippocampus—which mediates the hippocampal spatial behaviors and also modulates the HPA axis. The alteration in glucocorticoid receptor expression is rendered in the epigenome and then communicated through the generations (Zhang et al. 2013; Bludau et al. 2019). Specific to our focus, we notice that a genome-wide study of 12 humans with Post-traumatic stress disorder (PTSD) reveals that of the many genes epigenetically altered, at least 8 were associated with the olfactory sense—odorant receptor genes (Chen et al. 2016). It remains to be established how or to what extent olfaction might be altered and generationally communicated; however, it is compelling to consider the extent to which animals might convey olfactory information about their environment forward to future generations (Dias and Ressler 2014).

### It is never that simple—How does the body encode and communicate the absence of multiple needs at once to drive flexible neural signaling and behavior?

The above examples examine how the body encodes and communicates the absence of a single basic need, such as food or safety. However, in reality, internal states arise from drives to meet and balance multiple basic needs simultaneously. How do endocrine and neural systems juggle multiple needs to support homeostasis?

We begin by examining how *Eptesicus fuscus* (the big brown bat) balances food and safety needs. The big brown bat is a social species that relies on echolocation to mate, locate food, and reduce risk of predation (Chaverri et al. 2018; Fig. 3C). This species also exhibits seasonal behaviors, such as mating in the fall, hibernating in the winter, and gestating in the spring, which rely on seasonal tuning of the auditory system (Kurta and Baker 1990). Specifically, pregnant female bats must balance the drive to forage after months in torpor with the drive to remain safe and decrease the probability of predatory encounters until after giving birth. Electrophysiological recordings of single neurons in the inferior colliculus (IC), the main auditory center of bats (Wenstrup and Portfors 2011), suggest that decreased auditory sensitivity correlates with seasonal changes in gonadal hormone levels. These neural and endocrine changes may decrease female foraging behavior as bats rely on echolocation to locate their food. Decreased foraging in turn decreases the probability of predatory encounters and increases the likelihood of females surviving to give birth to their offspring. However, after giving birth, lactating mothers have an increased metabolic need, correlated with an increase in auditory neuron sensitivity, to promote foraging behavior and protect against predation (Miller et al. 2016).

How might internal state (i.e., changing hormonal levels) be communicated to tune auditory sensitivity? Mechanistic studies suggest that seasonal modifications of auditory sensitivity might be mediated by an interaction between endocrine and serotonergic signaling, as serotonin has been found to increase IC neuron latency in bats (Hurley and Pollak 2005). Sex steroid levels, such as those of estradiol, are highest during the late stages of pregnancy in female bats. At the same time, IC neurons become less sensitive due to increased first-spike latency. The timing of this auditory tuning is crucial for survival (Crichton and Krutzsch 2000). During the spring, however, after females give birth, they need to retune their auditory sensitivity. This is thought to allow them to hear their pups emitting long duration isolation calls during the first 2 weeks after birth and promotes food-seeking behavior. These changing behavioral needs after giving birth, coincide with a decrease in hormonal levels, and a peak in sensitivity within a subtype of duration-sensitive neurons (Monroy et al. 2011).

As Miller et al. note, findings in the big brown bat parallel research in songbirds, which could offer a potential way in which hormonal levels might be modulating serotonergic signaling to tune auditory processing. While a direct link between estradiol and serotonergic signaling has not been observed in the big brown bat, this link has been observed in breeding songbirds and rats (Biegon and McEwen 1982; Matragrano et al. 2012). Breeding songbirds display increased auditory response latency, which correlates with high estradiol levels, and an increase in the density of serotonin receptors in the main auditory pathway of birds (Caras et al. 2010; Matragrano et al. 2012). In addition to an increase in the density of serotonin receptors, more serotonin has also been observed in the auditory forebrain of breeding songbirds compared to those in nonbreeding conditions (Rodríguez-Saltos et al. 2018). This suggests that serotonergic responses that modulate the latency, and thus sensitivity, of neurons in the auditory forebrain needed for behaviors specific to each season could in turn be regulated by endocrine signaling that reflects both reproductive and feeding state (Hurley and Pollak 2005; Rodríguez-Saltos et al. 2018). In addition to changes in hormone levels, Miller et al. also suggested that future research on the big brown bat could help us understand how changes in endocrine signaling, seasonal light/dark cycle, and temperature influence auditory plasticity in order to encode and integrate the availability of multiple needs.

Neurophysiological studies often capture a snapshot of cellular or circuit activity in a short window of time. However, seasonal behaviors in particular, such as those displayed by the big brown bat, could serve as an exciting opportunity for neuroscientists to explore how the physiological state of the body, often communicated via the endocrine system over slower timescales, confers flexibility to sensory circuits often measured on faster timescales (Fig. 2 and Box 1). We are excited to see how researchers continue to explore what appears to be a gap in timing between these body-to-brain mechanisms.

Our second example highlights body-to-brain communication that occurs as animals regulate the basic needs of both food and sleep. Sleep is a universal behavior typically characterized by sustained periods of immobility and a reduced arousal threshold. There are likely to be many functions associated with sleep, including but not limited to memory consolidation (Haynes et al. 2015), synaptic homeostasis (Bushey et al. 2011), neurodevelopmental progression (Kayser and Biron 2016), and reproductive output (Potdar Sheetal et al. 2018). Furthermore, recent work has demonstrated that sleep reduces oxidative stress levels in the gut which in turn correlates to increases in lifespan (Vaccaro et al. 2020). The connection between gut function and sleep suggests a restorative role of sleep in relation to nutrient availability, and may start to provide insights into how body-to-brain interactions balance multiple needs at a time. Satiated animals can spend less time foraging in order to sleep longer and more deeply. Achieving this requires communication between physiological signals in the body and brain that detect the internal and external sensory environment in order to coordinate the appropriate behavioral response.

In *D. melanogaster* (fruit flies), the level of satiety affects responsiveness to external stimuli when flies are asleep via gut-to-brain communication (Titos and Dragana 2020; Vaccaro et al. 2020) (Fig. 3C'). Enteroendocrine cells are specialized cells in the gastrointestinal tract that synapse to and communicate with neurons via neuropeptides (Miguel-Aliaga et al. 2018). Specifically, enteroendocrine cells respond to protein consumption by producing and secreting the neuropeptide CCHamide-1 (Fujiwara et al. 2018). This peptide likely acts as a hormone, binding to CCHamide-1 receptors and increasing activity in dopaminergic neurons that innervate the mushroom body, a multisensory memory and learning center in the fly brain. It has been hypothesized that the fly mushroom body receives mechanosensory information, as is the case in other insects such as honeybees (Scheiner et al. 2001; Schröter and Menzel 2003; Li et al. 2020; Titos and Dragana 2020;). Dopaminergic mushroom body output neurons regulate sleep duration and sleep depth (Titos and Dragana 2020). While dopaminergic signaling commonly functions to increase arousal (Kume et al. 2005; Liu et al. 2012; Driscoll et al. 2020; Li et al. 2020), in this context, the activity of the dopaminergic neurons leads to decreased responsiveness to external stimuli and thus suppresses sensory arousability (Titos and Dragana 2020). In this way, the dopaminergic neurons integrate input from the gut about nutrient and sleep states with external sensory stimuli, allowing the internal state of satiety to modulate the flies’ responsiveness to stimuli while asleep.

This example highlights how changes in internal states communicate multiple basic needs by impacting behavior through a body-to-brain connection. While sleep has frequently been studied from a perspective of top-down control by the brain, these findings reveal an equally important reverse system of communication through which the gut impacts neural activity. The work suggests that animals that have had a high-quality meal can reap the restorative benefits of longer, deeper, uninterrupted sleep and a reduced need to be alert for foraging opportunities.

## To infinity loops and beyond: an outlook

Overall, we intend this perspective to offer an integrative framework for how internal states are represented and communicated within and between the body and brain, on multiple temporal and spatial scales. Together, the framework and studies we present here embody several themes in our understanding of internal state, detailed below.

First, the body plays a critical role in shaping internal state. The studies highlighted in this review add to the growing literature in both vertebrates and invertebrates showing that signals from the body can modify neural circuits at the very first stage of sensory processing (Root Cory et al. 2011; Sengupta 2013; Takeishi et al. 2020a, 2020b). The resulting changes at peripheral sensory neurons can lead to changes in the valence and salience of sensory inputs, altering processing in downstream circuits, and in turn modifying behavior. Studying sensory processing in the context of the body has shed light on key mechanisms that allow sensory circuits to flexibly respond to external stimuli. Furthermore, it is apparent that this framework can invite new perspectives on the function of basic needs, as suggested for instance in the work on the role of sleep in satiated versus hungry flies (Titos and Dragana 2020; Vaccaro et al. 2020).

A second core theme is that internal states are established and maintained by a distributed and highly interconnected system that consists of the many organs, tissues, and molecules within the body and brain. Additionally, this distributed network consists of dynamic structures that communicate and coordinate multiple functions between the body and brain across the many orders of space, and over the multiple timescales that an organism experiences. This is in contrast to the common structure–function framework, in which each biological structure is assumed to correspond to a static and single function. We welcome the added complexity in understanding the mechanisms underlying internal state from a distributed network perspective, which is in alignment with the growing understanding of distributed networks in neural systems (Hikosakaet al. 1989; Houk and Wise 1995; Dupre and Yuste 2017).

Third, we find utility in expressing the mechanisms of action as modules that are organized on a temporal axis across endocrine and neural systems, by analogy to behavior. To support experimental exploration across these axes, we offer the possibility of using the construct of modules of mechanisms across the body and brain that coordinate with each other to enable sequences of behavioral modules across longer timescales than those typically considered in neuroscience, including (but not limited to) seasonal, lifetime, and generational. This approach can help to identify gaps and offer new possibilities for future studies. We invite you to apply this framework to your own systems of study and predict what other body–brain systems can be explored using this approach.

We find that multidirectional body–brain communication is an exciting framework by which to study internal state, and acknowledge that this integrative approach comes with many challenges. For instance, selecting sampling rates that accurately describe the dynamic shifts and rhythms of signaling across different body systems and the brain is a difficult task. Thus, we are excited that the completion of this Perspective coincides with a re-emerging focus within the broader neuroscience community on the body’s role in understanding internal state. Importantly, this re-emerging focus is accompanied by an urgency and ongoing discussions to develop tools that support the exploration of multidirectional body–brain communication pathways.

Our ideas also relate to important theories developed over the last century—including enactivism, embodied cognition, and cybernetics—which suggest that cognition fundamentally depends on body–brain interactions (Norbert 1961; Cisek 1999; Cisek and Kalaska 2010; Clark 2013; Pezzulo and Cisek 2016; Schwartz 2016; Brette 2019; Cisek 2019; Parker et al. 2020; Teufel and Fletcher 2020). The timeliness of our Perspective further coincides with recent funding announcements focused on internal state and interoception from two influential research institutes, the National Institutes of Health and Janelia Research Campus, as well as a special issue exploring the field of interoception in *Trends in Neuroscience* (Berntson and Khalsa 2021; Chen et al. 2021; Petzschner et al. 2021). We intend for this Perspective to build upon these reviews and provide evidence that contributions from a diverse array of model and non-traditional model organisms will be important to advancing our understanding of how dynamic changes are communicated between the body and brain.

## Notes

From the Symposium “Spatiotemporal dynamics of animal communication” presented at the Annual Meeting of the Society for Integrative and Comparative Biology, Virtual meeting, January 7, 2021.

## New data and availability of data

Labanotation of Bhangra phrase was generated in LabanWriter by Valarie Williams in support of this research. The data underlying this article are available in the article.
